# P-2127. Frequency, Interval, and Patient Factors Associated With Recurrence of Disseminated Cutaneous Coccidioidomycosis

**DOI:** 10.1093/ofid/ofae631.2283

**Published:** 2025-01-29

**Authors:** Nathan Chow, Janis E Blair

**Affiliations:** Mayo Clinic School of Graduate Medical Education, Scottsdale, Arizona; Mayo Clinic Hospital, Phoenix, Arizona

## Abstract

**Background:**

Coccidioidomycosis (cocci) is a fungal infection caused by *Coccidioides* and a common cause of community acquired pneumonia in endemic regions. Disseminated cocci occurs in ≤ 1% of persons with cocci, and among these, disseminated cutaneous coccidioidomycosis (DCC) is estimated to occur in 15 to 67% of cases, however little data exists to describe its frequency and timing of recurrence. Limited small studies show recurrence rates from 17 to 50% and often do not distinguish skin dissemination from other soft tissues. Our aim was to determine the frequency, timeframe and risk factors for relapsed DCC.

**Methods:**

Potential cases of disseminated cocci were identified by ICD-10 codes 38.3, 38.7, 38.9 (& ICD-9 equivalents) and keyword-based search of pathology reports from 1/1/2008 - 3/31/2024. We included patients with proven or probable DCC. We excluded patients with primary cutaneous cocci or reactive rash. We performed a retrospective review to identify episodes of recurrence, comorbidities, and antifungal regimen.

**Results:**

We identified 44 proven and 3 probable cases of DCC (33 males, 14 females), median age 48 years, 66.0% white, 21.3% black, 5.4% Hispanic. 18 (38.3%) were immunosuppressed and 6 (13.6%) had diabetes mellitus. Median follow-up duration was 68 months; 12 (25.5%) patients had ≥1 recurrence, 7 (14.9%) of whom relapsed ≥2 times. Median interval off of antifungals until recurrence was 14 months (range: 1 - 96). 3 (6.4%) patients had recurrence within three months of diagnosis, and 6 (13.0%) and 11 (23.9%) had a recurrence by the 1st and 5th follow-up year, respectively. 21 (87.5%) recurrences were at the same site as a prior occurrence, 4 (18.2%) occurred on antifungal therapy. There were no significant differences in recurrence rates by race or diabetes. Of the 18 immunosuppressed cases, there was 1 (5.6%) recurrence, though 14 (77.8%) continued antifungal therapy throughout follow-up.

**Conclusion:**

Once antifungal treatment was discontinued, recurrence of disseminated cutaneous cocci was common, and among those whose DCC relapsed, multiple relapses were noted. Longer follow up is needed to better characterize the risk of long-term recurrence.

**Disclosures:**

All Authors: No reported disclosures

